# Lumpfish, *Cyclopterus lumpus*, distribution in the Gulf of Maine, USA: observations from fisheries independent and dependent catch data

**DOI:** 10.7717/peerj.17832

**Published:** 2024-08-14

**Authors:** Elizabeth A. Fairchild, Sophie Wulfing, Easton R. White

**Affiliations:** Biological Sciences, University of New Hampshire, Durham, NH, United States of America

**Keywords:** Range shift, Aquaculture, Water temperature, Lumpfish, Data limited species, Species range shift, Gulf of Maine, Fisheries

## Abstract

The Gulf of Maine (GoM) is one of the fastest-warming parts of the world’s oceans. Some species’ distributional shifts have already been documented, especially for commercially-important species. Less is known about species that are not currently exploited but may become so in the future. As a case study into these issues, we focus on lumpfish (*Cyclopterus lumpus)* because of the recognized and timely need to understand wild lumpfish population dynamics to support sustainable fisheries and aquaculture developments. Using occurrence data from five different fisheries-dependent and independent surveys, we examined lumpfish distribution over time in the GoM. We found that lumpfish presence was more likely in Fall and correlated with deeper waters and colder bottom temperatures. Since 1980, lumpfish presence has increased over time and shifted north. Given a limited set of data, these findings should be interpreted with caution as additional work is needed to assess if the actual distribution of lumpfish is changing. Nevertheless, our work provides preliminary information for resource managers to ensure that lumpfish are harvested sustainably for use in emergent lumpfish aquaculture facilities.

## Introduction

Climate change is expected to change the distribution of many marine species, primarily with poleward shifts (Perry et al., 2005; Campana et al., 2020). The Gulf of Maine (GoM) is an interesting case study applicable to this issue as the area is one of the fastest warming bodies of water on the planet (Pershing et al., 2015). Yet, most work on species distribution shifts has focused on species of commercial interest given funding and data availability (Pershing et al., 2015; Free et al., 2019; Goode et al., 2019; Fredston-Hermann et al., 2020). Evaluating changes over time to unexploited species can be telling of ecosystem changes (*e.g.*, marine historical ecology; Engelhard et al., 2016) yet difficult to do because these species are not usually the target of long-term fisheries surveys and are data-limited. Often these data-limited species appear as bycatch but only in small numbers. However, it is possible to combine multiple data sets using different gear types to ensure adequate sample sizes. For example, fisheries-independent trawl surveys, recreational fish surveys, and citizen science dive surveys were aggregated and modeled to predict temporal changes in rockfish distribution in Puget Sound, WA (Tolimieri et al., 2017). Coupling long term catch information with abiotic and biotic variables can provide insight into how these drivers affect ecological communities (*e.g.*, Hampton et al., 2013; Rogers et al., 2019).

While well-studied in other areas within its range, lumpfish (*Cyclopterus lumpus*) is an example of a species with limited data in the GoM, and one that may be exploited in US waters in the near future for its use as a cleanerfish in northern New England salmonid farming operations (J Ford, Cooke Aquaculture, pers. comm., 2023). Currently, lumpfish is not a regulated species in US waters, therefore, no fishery management plan exists for them. However, in Canada, lumpfish are listed as Threatened due to steep population decreases most likely caused by fishing (DFO, 2021). As they become exploited in US waters, lumpfish will need to be managed and, to do so sustainably, resource managers need to be informed of baseline data on fish biomass, occurrence, and distribution.

Lumpfish are distributed in temperate waters (3−10 °C; Rodríguez-Rey & Whittaker, 2023) in both the eastern and western Atlantic Ocean at high latitudes. In the eastern Atlantic, lumpfish are found in the Barents Sea, around Iceland and Greenland, and as far south as Spain and Portugal (Stein, 1986). In the western Atlantic, lumpfish range from Hudson Bay and Labrador, Canada southwards along the US eastern seaboard to New Jersey (Stein, 1986). Lumpfish are characterized as semi-pelagic but inhabit both the pelagic and demersal zones and undertake diel vertical migrations depending on the season (Kennedy et al., 2016). In Icelandic waters, adult lumpfish typically are found at depths <300 m but have been caught as deep as 518 m (Kennedy et al., 2016). As adults, lumpfish tend to live offshore, preferring cooler (Iceland: 0−6 °C, Kennedy et al., 2016; Barents Sea: 4−7 °C, Eriksen, Durif & Prozorkevich, 2014) waters; however, during the spring spawning season, adults migrate inshore to the coastlines (Goulet, Green & Shears, 1986; Collette & Klein-MacPhee, 2002). Larval lumpfish typically are found in shallower coastal waters, but can occur offshore too (*i.e.,* Barents Sea, Eriksen, Durif & Prozorkevich, 2014). Juvenile lumpfish prefer slightly warmer waters than adult fish (Norway: 6−7 °C, Mortensen et al., 2020; Barents Sea 5−7 °C, Eriksen, Durif & Prozorkevich, 2014). With increasing water temperatures, the probability of occurrence for lumpfish declines (Rodríguez-Rey & Whittaker, 2023).

In the USA, lumpfish have never been exploited, and as a result little is known about lumpfish populations in US waters (Collette & Klein-MacPhee, 2002). Published information is limited to Great Bay Estuary, NH (Rackovan & Howell, 2017) and Schoodic Peninsula, ME (*e.g.*, Moring, 1989; Moring, 2001). Remaining population information is inferred from studies throughout the other areas of their range. Semi- pelagic adults move inshore to rocky coasts to spawn from March to May in the southwestern GoM, and May to June along the northeast Maine coast (Cox & Anderson, 1922; Goulet, Green & Shears, 1986; Collette & Klein-MacPhee, 2002). Females spawn two sticky, demersal egg masses and then move offshore, while the males stay to guard and tend the eggs until hatching, which occurs after approximately six to eight weeks, depending on water temperatures (Cox & Anderson, 1922; Collins, 1978; Goulet, Green & Shears, 1986; Martin-Robichaud, 1991; Kennedy, 2018). Juvenile lumpfish leave the nest area in the early summer and are highly associated with macroalgae, either in tidepools or in the upper 0.5 m of the water column (Daborn & Gregory, 1983; Moring, 1989; Rackovan & Howell, 2017), where they prey on small invertebrates including amphipods, copepods, isopods, and even small fish larvae (Moring, 1989; Tully & O’Ceidigh, 1989; Davenport & Rees, 1993). They depend on seaweed for transportation as it passively drifts, providing protection from predators and an increased food source (Vandendriessche et al., 2007). Lumpfish grow quickly in their first year of life compared to other cold-water marine fishes, reaching approximately 35 to 70 mm in total length (TL; Martin-Robichaud, 1991). During this time, most of their energy is diverted towards growth since the fish cling to algae and wait for prey to pass by (Brown, 1986; Killen, Brown & Gamperl, 2007). At age 1+, lumpfish become mostly pelagic and move into deeper waters (Blacker, 1983). Lumpfish can live up to approximately 10 to 15 years. In the wild, males reach sexual maturity in two to three years while it takes females three to four years (Albert et al., 2002; Hedeholm, ME & Grønkjær, 2014). While GoM lumpfish are considered part of a western Atlantic lumpfish stock unit, composed of US and Canadian fish (Whittaker, Consuegra & Garcia deLeaniz, 2018), they are distinct from the Canadian populations (Langille et al., 2023; Langille et al., 2024).

Despite the few GoM-based lumpfish studies, numerous long-term state and federal surveys that include lumpfish catch data exist which could be used to provide more information about occurrence of lumpfish in the GoM. Therefore, our study goals were to: (1) aggregate GoM lumpfish catch data and characterize lumpfish distribution, and (2) determine if and how water temperature may have affected lumpfish distribution over time. We hypothesized that lumpfish distribution would be correlated to water temperature both temporally and spatially, shifting northeast with increases in water temperature.

## Materials & Methods

### Lumpfish source data

We acquired historic information on lumpfish caught between 1963 (the start date depends on the dataset) and 2021 from Maine (ME), New Hampshire (NH), Massachusetts (MA), and the Northeast Fisheries Science Center (NEFSC) fisheries-independent surveys and from the fishery-dependent NEFSC observer program (Table 1). Except for the data collected by the observer program, all data are publicly available. While each data set we accessed was unique, each contained at a minimum date, location, and depth for each fish caught. Most non-observer data included bottom temperature, and most data sets contained fish size information (individual length, individual weight or batch weight). Gear types and sampling methods varied as noted below.^1^ 1Portions of this text were previously published as part of a preprint (https://ecoevorxiv.org/repository/object/6888/download/13246/).

**Table 1:** Lumpfish catch data from fish surveys in the Gulf of Maine.

**Location**	**Agency**	**Survey name**	**Gear used**	**Dates**	**# Tows**	**Total lumpfish**	
ME/NH	Maine Dept of Natural Resources	Maine-NH Inshore Trawl Survey	Bottom trawl	2000–2021	3,579		1,357
NH	New Hampshire Fish & Game Dept	Estuarine Survey of Juvenile Fish	Seine	1997–2021	46		104
MA	Massachusetts Division of Marine Fisheries	Bottom Trawl Survey	Bottom trawl	1978–2021	8,087		120
Federal Waters	NEFSC	Bottom Trawl Survey	Bottom trawl	1963–2021	40,084		649
Federal Waters	NEFSC	Observer Data	Multiple	1989–2021	10,122		9,910

*Maine Department of Marine Resources (ME DMR) Maine-NH Inshore Trawl Survey*: A stratified random survey, separated into four depth strata and five geographic regions along the coast of Maine and New Hampshire, ranging from 5 m at the shallowest along the coast out to 19.3 km (12 miles). A total of 120 stations were randomly selected for sampling for each spring survey, then resampled again in the fall with a modified shrimp net with a 2.5 cm codend liner towed for 20 min. All catch was sorted by species, total weights taken per species, and individual lengths measured (total length (TL) for lumpfish). Bottom and surface temperature and salinity were measured during each tow with a conductivity-temperature-depth (CTD) profiler (Sea-Bird Electronics™ 19plus SEACAT) attached to the starboard door wire. Survey methods are reported by Sherman, Stepanek & Sowles (2005).

*New Hampshire Fish and Game Department (NH F&G) Estuarine Survey of Juvenile Fish*: A monthly seine survey at 15 fixed stations in Hampton-Seabrook and Great Bay Estuaries in NH, occurring June through November each year. One haul per site per month was conducted at low tide in waters <2 m using a seine measuring 30.5 m long by 1.8 m high with 6.4 mm mesh. All catch was sorted by species and individual lengths taken. Surface temperature and salinity were measured, and bottom substrate type documented. Detailed survey results are documented in New Hampshire Fish and Game Department (NHF&G) (2020).

*Massachusetts Division of Marine Fisheries (MA DMF) Bottom Trawl Survey*: A stratified random bottom trawl survey in five regions over six depth zones ranging from <9 m to >55 m in both spring and fall in MA state waters. Approximately one station per 19 square nautical miles was sampled by a 20-minute tow taken with a $ \frac{3}{4} $ size North Atlantic type two seam otter trawl with a 6.4 mm codend liner. All catch was sorted by species and total weight per species per tow was recorded. Bottom temperature was measured continuously during each tow with a data logger (Onset HOBO Water Temperature Pro v2 Data Logger) attached to the net’s headrope. Detailed information is found in Camisa, Manfredi & Glenn (2020).

*NEFSC Bottom Trawl Survey*: A spring and fall stratified random bottom trawl survey occurring most years (1963–2021), but with other seasons sampled sporadically in the past (1991–1995 summer GoM survey; 1992–2007 winter bottom trawl survey). Due to the timespan of this survey, bottom trawl gear specifications and protocols changed slightly over the years. Generally, the survey occurred from North Carolina to Nova Scotia, but occurred in some years as far south as Florida. While our study focuses on lumpfish distribution in the GoM, we included all lumpfish catch from this dataset. Surveyed areas ranged in depth from 18 to 366 m with >300 tows made per survey. All catch was sorted by species and most individuals were weighed and lengths measured. Bottom temperature was measured at the deepest observation at each sampling site that fell within 10 m of the reported water depth. Survey details can be found at: https://www.fisheries.noaa.gov/inport/item/22557.

*NEFSC Observer Data*: Fishery-dependent data collected by observers on board commercial fishing boats throughout the year and throughout the GoM from multiple fisheries, but lumpfish caught mostly when groundfish, herring, and sea scallops were targeted. Gear types varied but included standard bottom trawl, midwater trawl, paired midwater trawl, gillnet (both drift-sink and fixed), and scallop dredge (Table 2). Data collected included location, depth, gear type, and for a subsample of fish, lengths were measured. For those fish measured, individual fish weights were also recorded, otherwise fish were batch weighed and sample size not recorded. For some trips, surface temperature was recorded. Data are available by request directly from the Observer Program: https://www.fisheries.noaa.gov/new-england-mid-atlantic/fisheries-observers/fisheries-monitoring-operations-northeast.

**Table 2:** Number of lumpfish caught 1989–2021 by various commercial gear types as documented by fisheries observers. Gear type descriptions are federal observer program codes.

**Gear type – observer data**	**Total lumpfish**
Trawl, otter, bottom, fish	8,885
Gill net, fixed or anchored, sink, other/unspecified	640
Trawl, otter, midwater paired	103
Dredge, scallop, sea	58
Trawl, otter, midwater	54
Trawl, otter, bottom, shrimp	48
Trawl, otter, bottom, haddock separator	47
Gill net, drift-sink, fish	46
Trawl, otter, bottom, twin	10
Trawl, otter, bottom paired	6
Longline, bottom	5
Pot/trap, lobster offshore nk	2
Trawl, otter, bottom, Ruhle	2
Dredge, other/nk species	2
Trawl, shrimp, twinned	1
Handline	1
All gears combined	9,910

### Data cleaning and analysis

We combined datasets to include tow data such as latitude and longitude (decimal degrees) of catch, date, season, and depth of catch when present in the data. We included environmental variables, such as bottom and surface water temperature (°C), air temperature (°C), and bottom and surface water salinities (ppt), where available. Catch data included number of lumpfish caught, fish length (TL), as well as several fish weight (kg) categories. Some datasets reported weights of individual fish, whereas other datasets aggregated the weight of all lumpfish in each tow. We used the most precise weight data when available. For fish lacking individual weights, we estimated weights using the Bayesian length-weight relationship: Weight = *α* Length ^β^. These were calculated using alpha and beta values of *α* = 0.02630 (0.01101 - 0.06285) and *β* = 2.99 (2.77 - 3.21) (Froese, Thorson & Reyes, 2014). From these calculations, survey length-frequency distributions, and other studies (Collins, 1979; Albert et al., 2002; Eriksen, Durif & Prozorkevich, 2014), we separated lumpfish into life history categories: Young-of-Year (age 0; <7 cm TL), Juvenile (age 1; 7–17 cm TL), and Adult (age 2+; >17 cm TL).

To better understand the range shifts of lumpfish, we mapped the distribution of lumpfish over each season, as well as the bottom temperature (where available) where each lumpfish was located when caught. We defined seasons as winter (December through February), spring (March through May), summer (June through August), and fall (September through November). Using the NEFSC bottom trawl surveys, which included effort, we built a series of generalized linear models with Binomial error distributions to understand potential correlates, including bottom temperature, of lumpfish presence *vs* absence in the catch over time. We accounted for spatial autocorrelation with a smoothing function of latitude and longitude using an exponential decay for correlation. We verified model assumptions by visually inspecting residual plots.

## Results

**Figure 1:**
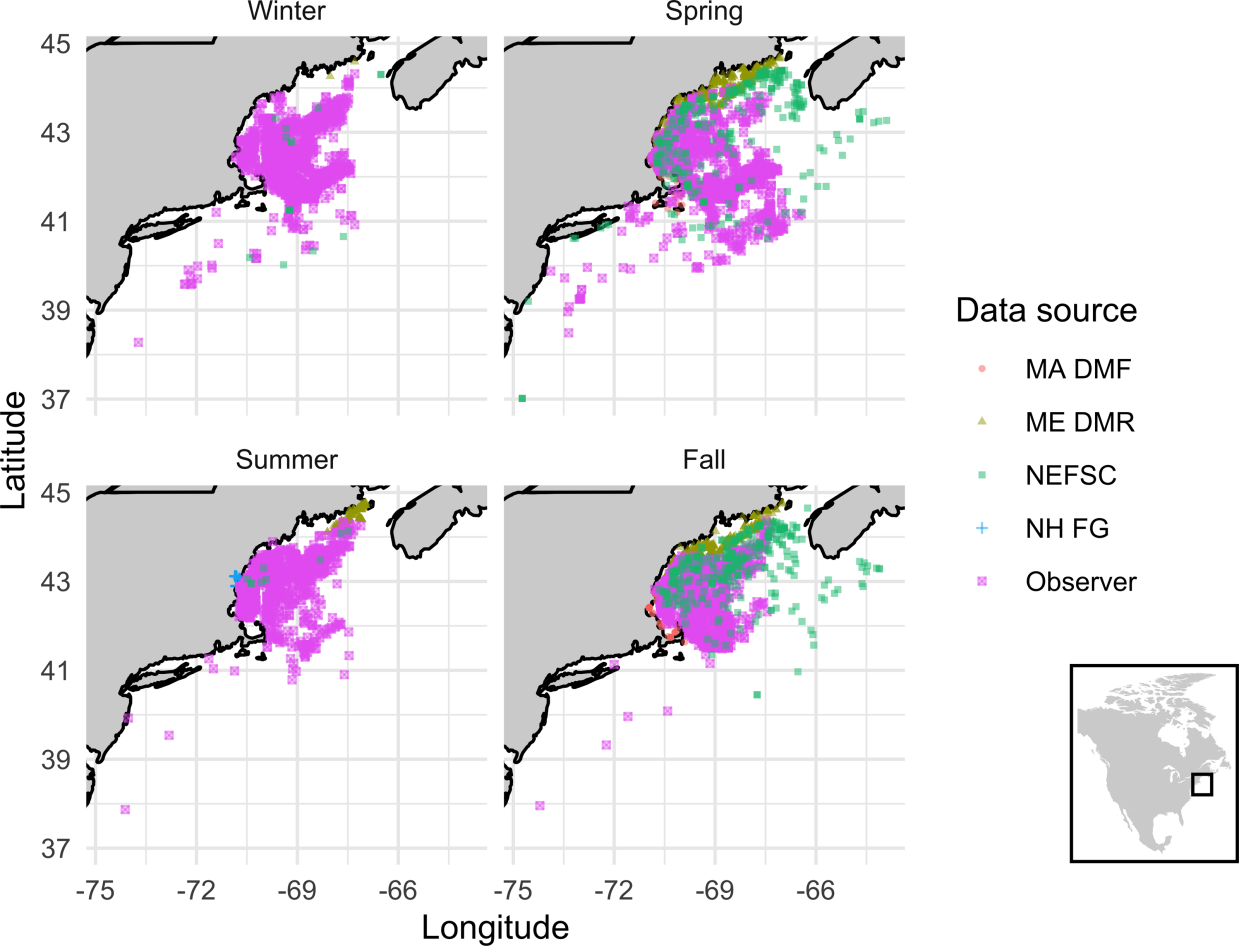
Seasonal lumpfish catch in the Gulf of Maine. Seasonal lumpfish catch in the Gulf of Maine from state and federal surveys spanning 1963–2021. Winter = Dec–Feb; Spring = March–May; Summer = June–Aug; Fall = Sept–Nov. See Table 1 for additional information about data sources.

Across the five datasets, we identified over 12,000 instances of lumpfish being caught across the five datasets, including 9,910 in the 1989–2021 NEFSC Observer data (Table 1). Most of the datasets only indicate positive catch records and not true catch-per-unit effort (although we explore the NEFSC Bottom Trawl data further below to address this point). There was enormous variability in the spatial and temporal scales of catch between the datasets (Fig. 1, Table 1). The NEFSC Observer and Bottom Trawl programs caught lumpfish over the largest range, which is in line with expectations given sampling protocols due to the other dataset’s fishing efforts focused nearshore (Fig. 1). NEFSC Observer data were the most consistent throughout the year. The NEFSC Bottom Trawl and most state surveys were conducted only during spring and fall. Most lumpfish were caught by various types of trawl gear or gill nets (Table 2).

**Figure 2:**
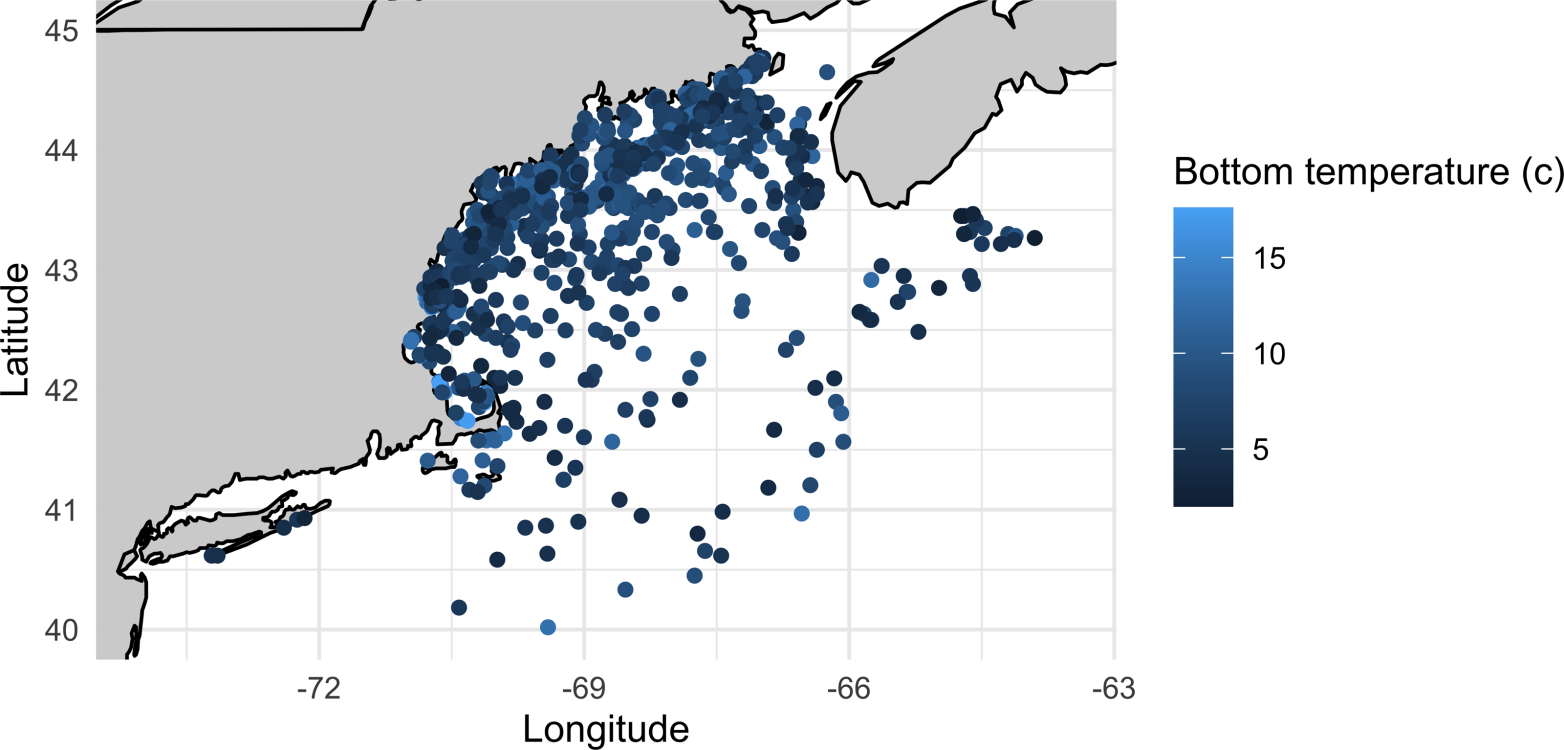
Bottom temperature (°C) where lumpfish were caught. Bottom temperature (°C) where lumpfish were caught in the Gulf of Maine from 1963–2021. Bottom temperature only was recorded for surveys conducted by MA DMF, ME DMR, and the NEFSC.

**Figure 3:**
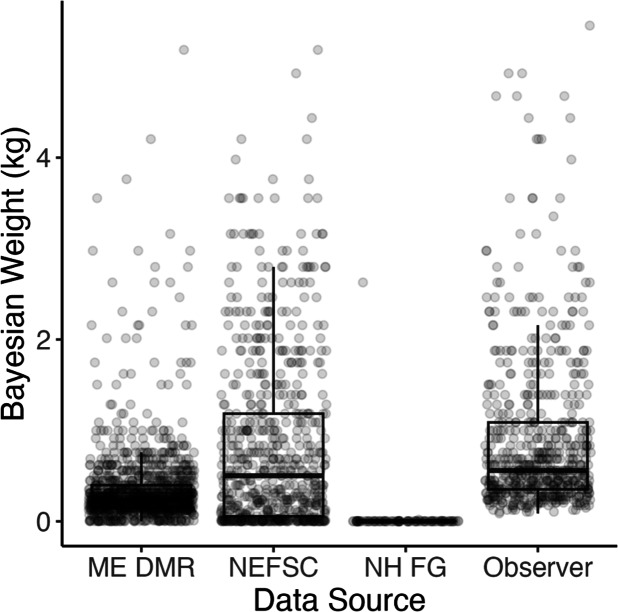
Bayesian weights (kg) of lumpfish. Mean calculated Bayesian weights (kg) of lumpfish caught by source. MA DMF data are not included as lumpfish lengths were not reported.

**Figure 4:**
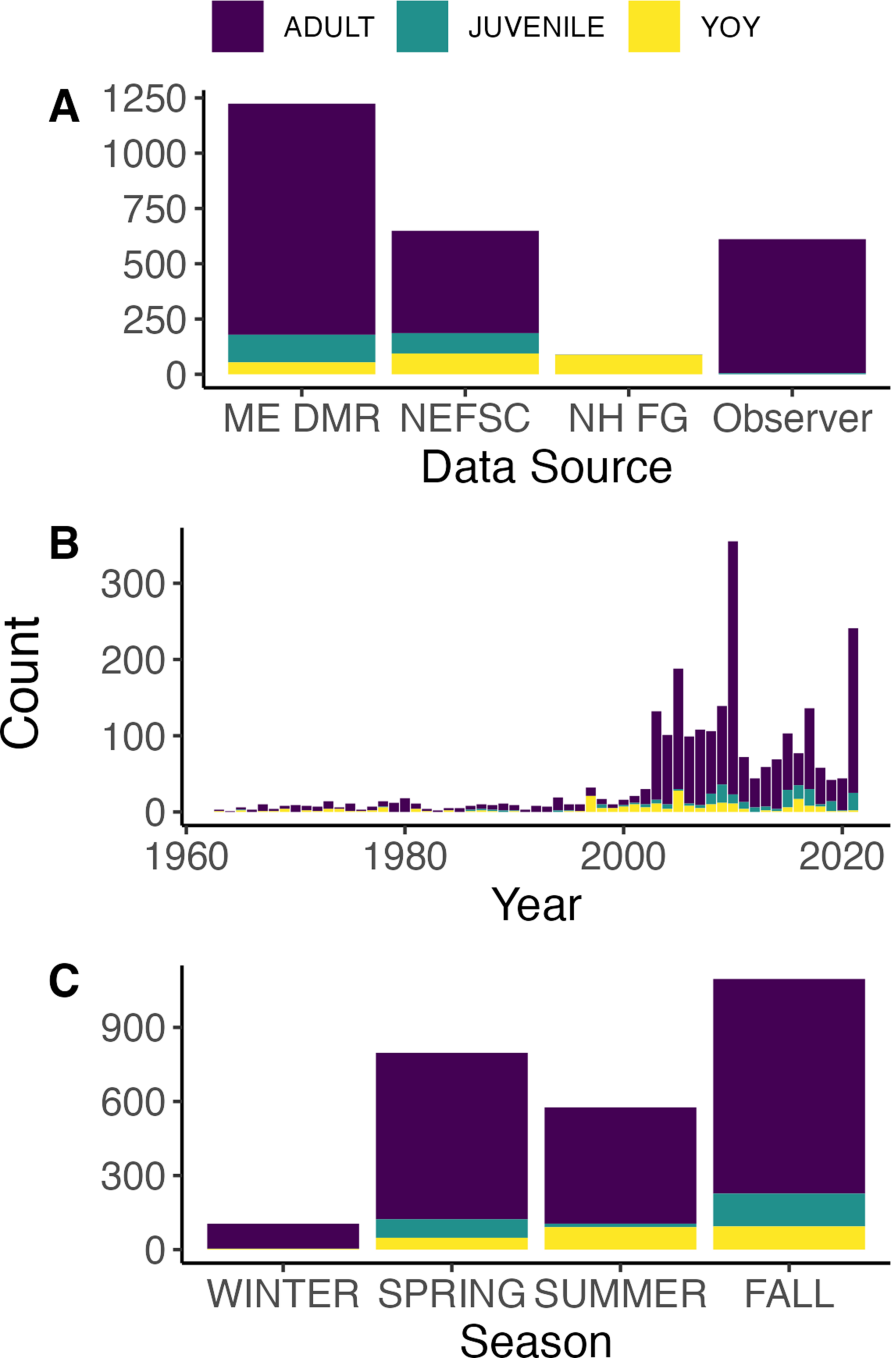
Timeseries of catch by age. Number of lumpfish caught by year in the Gulf of Maine from 1963–2021 from all state and federal surveys depicted by (A) age, (B) proportion, and (C) season. MA DMF data are not included as lumpfish age data could not be calculated.

**Figure 5:**
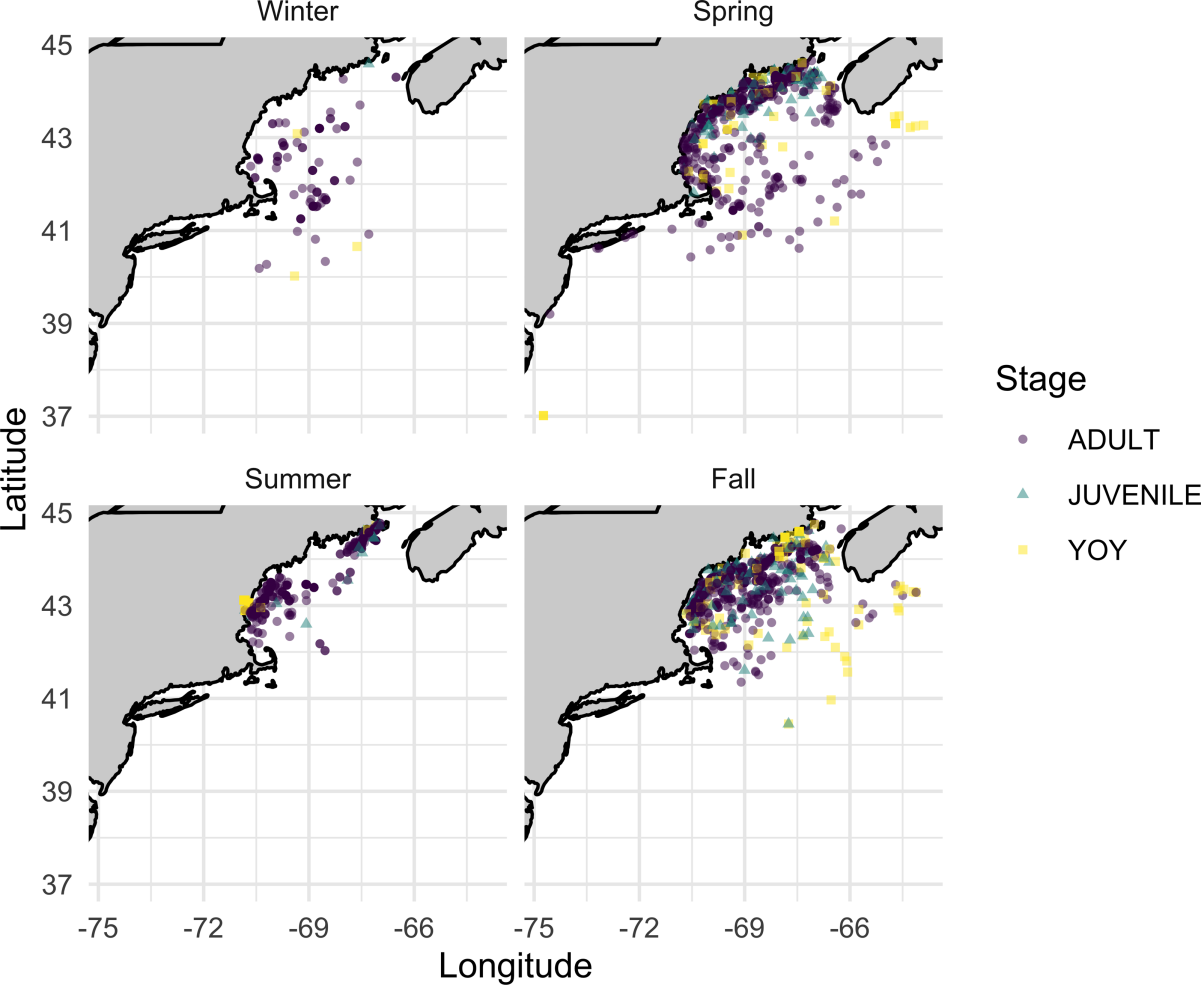
Distributions of lumpfish by age. Distributions of (A) YOY, (B) juvenile, and (C) adult lumpfish caught in the Gulf of Maine from 1963–2021 from all state and federal surveys. MA DMF data are not included as lumpfish age data could not be calculated.

Lumpfish were caught at bottom temperatures ranging from 2  °C to 17.6 °C and at depths ranging from 2 m to 393 m (Fig. 2). Most fish caught were adult individuals, however, NH F&G surveys only caught YOY fish (Figs. 3–4). NH F&G surveys occurred closer to shore and only in estuaries, which often act as nursery habitats for lumpfish (Rackovan & Howell, 2017). Further, NEFSC Observer surveys rarely caught juvenile and YOY individuals due to gear selectivity as they use standard legal fishing gear, which have larger mesh sizes than the fishery-independent surveys (Fig. 4A). Adult lumpfish were caught throughout the GoM whereas juvenile and YOY fish were mostly caught inshore (Fig. 5). Adult individuals were also the age group caught the most throughout each of the seasons, with very few juvenile and YOY individuals caught in the winter season (Figs. 3–4).

**Figure 6:**
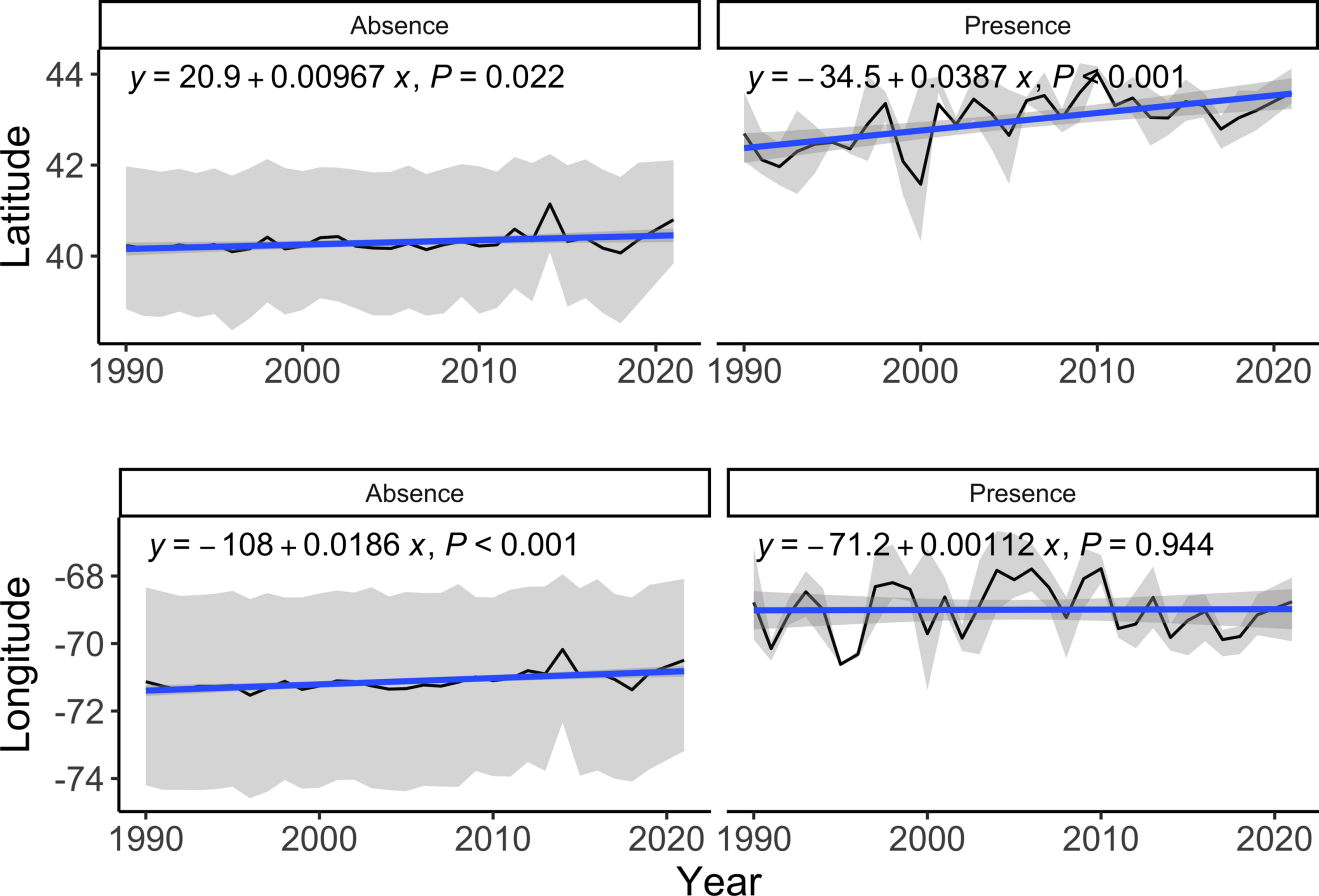
Time series of latitude and longitude of surveys where lumpfish were caught. Time series of latitude and longitude of surveys where lumpfish were either absent or present in the NEFSC BT surveys from 1980–2021.

Using presence-absence data from the NEFSC BT surveys (which also included effort), we assessed which covariates might affect lumpfish presence in catch. While accounting for spatial autocorrelation, we found that lumpfish presence increased over time (*b* = 0.029, *p* < 0.0001). Here, the estimate indicates a 0.037 increase in log-odds (or 1.038 odds) for each increase in year. Lumpfish presence was also less likely in spring (b =−1.41, *p* < 0.0001) compared to fall surveys. We also found lumpfish presence was more likely in deeper (*b* = 0.006, *p* < 0.0001) and colder bottom depths (b =−0.39, *p* < 0.0001). Although all the variables were significant, the effect size for depth was relatively small compared to the other parameters. Using a separate set of analyses, we found that the yearly latitude of lumpfish presence in catch increased over time (*b* = 0.039, *p* < 0.001). In other words, lumpfish presence was more likely at higher latitudes over time (Fig. 6). In addition, lumpfish presence did not shift longitudinally over time (*b* = 0.001, *p* = 0.944).

## Discussion

Although lumpfish are relatively uncommon in catch data in the GoM, we were able to identify 12,140 instances of catch when combing five disparate datasets spanning five decades (Table 1). Our findings are in line with past studies (*e.g.*, Collette & Klein-MacPhee, 2002) showing that adult lumpfish inhabit the GoM broadly both temporally and spatially, whereas YOY and juvenile lumpfish, for the most part, are found nearshore (*e.g.*, Moring, 2001; Rackovan & Howell, 2017). The predominance of younger fish nearshore during summer and fall, as evidenced from NH F&G and ME DMR surveys, indicates lumpfish spawn and tend egg nests in these waters. Surprisingly, these life stages were also found offshore indicating that larvae drift offshore and adults may be spawning offshore too, similar to Barents Sea lumpfish populations (Eriksen, Durif & Prozorkevich, 2014; Fig. 5C). Multiple studies have shown seasonal inshore-offshore movements of adult lumpfish associated with spawning, both in the GoM (Davenport, 1985) and elsewhere (*e.g.*, Iceland: Kennedy et al., 2014; Kennedy & Ólafsson, 2019; Norway: Mitamura et al., 2012). However, lumpfish spawning and completing the life cycle offshore is not well documented.

We show clear differences in catch composition given differences in sampling design and between the five monitoring programs. There were differences in the catch composition for nearshore *versus* offshore surveys, by season, and with gear type. Most gear used in the surveys was bottom gear fished during the daytime. Because lumpfish exhibit diel vertical behavior occupying the demersal zone during the daytime, gears used by the monitoring programs (*e.g.*, bottom trawl net, gillnet, dredge) are effective for characterizing lumpfish distribution (Eriksen, Durif & Prozorkevich, 2014; Kennedy et al., 2016). However, since the survey gears differed by mesh sizes (thus selected different sized lumpfish), survey depths (estuarine, nearshore, offshore), and areas (by state or federal waters), all catch data should be considered to more completely depict lumpfish distribution across depths, space, seasons, and life history stages in GoM waters (Kennedy & Jónsson, 2017).

We found some, albeit weak, support for our hypothesis that lumpfish distribution has shifted northeast in the past few decades (Fig. 6). Lumpfish were generally caught at deeper depths and colder bottom temperatures (Fig. 2). These results should be interpreted with caution as it is not clear how the distribution is shifting, given the limited data, despite significant increases in latitude. The GoM is one of the fastest warming bodies in the world (Pershing et al., 2015; Balch et al., 2022; GMRI, 2024). Lumpfish are a cold-adapted species, so a distributional shift with temperature aligns with their life history characteristics (Collette & Klein-MacPhee, 2002) and has been projected for more northern lumpfish populations (Rodríguez-Rey & Whittaker, 2023). Within the GoM, there have been other accounts of species moving in relation with warming waters (Le Bris et al., 2018; Friedland et al., 2023). Past work has shown that some species may see range expansions (*e.g.*, spiny dogfish and American lobster) while other more northern species (*e.g.*, Acadian redfish, American plaice, Atlantic cod, haddock, and thorny skate) will experience range constrictions (Kleisner et al., 2017). For lumpfish, the GoM is towards the southern end of their range and, as it continues to warm, will likely become increasingly less suitable for lumpfish (Rodríguez-Rey & Whittaker, 2023). Additional work is needed to understand how changes in other oceanographic variables (*e.g.*, nitrate, salinity, productivity; Rodríguez-Rey & Whittaker, 2023) may interact with temperature increases (Pershing et al., 2021) and changes to fishing pressures to affect GoM species in the future.

Over the course of five decades of data, there were changes in sampling protocols within and across our five datasets. Also, effort data were not associated with all the datasets, which limited our ability to conduct more detailed analyses. Future work could use more sophisticated approaches (Fletcher Jr et al., 2019) to combine these disparate datasets more formally. We had associated metadata (*e.g.*, bottom temperature, salinity) for only some of the datasets we used. Future work could collate and combine similar types of metadata from other available sources. Further, consideration could be given to sea temperature data as bottom temperature may not be the strongest driver to predict marine species’ shifts, but rather a temperature composite of the water column (Friedland et al., 2023). We also did not address how large perturbations (*e.g.*, storm events) may affect population trends differently than long-term oceanographic changes. We also did not study how behavioral responses by harvesters may change with seafood demand. There could also be additional work to understand how socio-ecological dynamics may interact with extreme events (White & Wulfing, 2023) to affect lumpfish. Finally, we only examined linear trends in lumpfish catch and distribution over time. Future work could examine how population dynamics may be changing in nonlinear ways (Bruel & White, 2021; Boënnec, Dakos & Devictor, 2024). Given predicted demand for lumpfish in aquaculture, our findings highlight the need for further research on the status of lumpfish in the GoM. If exploited, proper management must ensure lumpfish are harvested responsibly and overfishing prevented, especially because adults return to the same spawning areas (Davenport, 1985; Kennedy et al., 2014) at the same times (Kennedy & Ólafsson, 2019) each year and discrete lumpfish populations exist (Langille et al., 2023; Langille et al., 2024).

## Conclusions

Lumpfish in the USA present a rare opportunity to understand the population dynamics of a species that has never been exploited and provide information for sustainable harvesting practices. We found limited support for our hypothesis that lumpfish occurrences are shifting northeast along with increases in temperature. We also found that the probability of catching lumpfish increased over time and there were higher catches during Fall, at greater depths, and colder bottom temperatures. We hope this paper provides a foundation for future work on lumpfish, geared towards this emergent aquaculture sector, including lumpfish movements, genetic structure, stock assessments, and latitudinal population effects.

## Additional Information and Declarations

### Data Availability

The following information was supplied regarding data availability: The datasets are available at GitHub and Zenodo: - https://github.com/swulfing/TeamLump
- White, E. (2024). Gulf of Maine lumpfish. Zenodo. https://doi.org/10.5281/zenodo.12723476
